# A novel DNA double-strand breaks biosensor based on fluorescence resonance energy transfer

**DOI:** 10.1186/s40824-023-00354-1

**Published:** 2023-02-17

**Authors:** Jung-Soo Suh, Tae-Jin Kim

**Affiliations:** 1 Department of Integrated Biological Science, Pusan National University, Pusan, 46241 Republic of Korea; 2 Department of Biological Sciences, Pusan National University, Pusan, 46241 Republic of Korea; 3 Institute of Systems Biology, Pusan National University, Pusan, 46241 Republic of Korea

**Keywords:** DNA double-strand breaks, γH2AX, Fluorescence resonance energy transfer, Biosensor, Live-cell imaging

## Abstract

Revealing the spatiotemporal behavior of DNA double-strand breaks (DSBs) is crucial for understanding the processes of DNA damage and repair. Traditionally, γH2AX and DNA damage response (DDR) factors have been used to detect DSBs using classical biochemical assays, such as antibody-based immunostaining. However, a reliable method to visualize and assess DSB activity real-time in living cells is yet to be established. Herein, we developed a novel DNA double-strand breaks biosensor (DSBS) based on fluorescence resonance energy transfer (FRET) by employing the H2AX and BRCT1 domains. By applying FRET imaging with DSBS, we show that DSBS specifically reacts to drug- or ionizing radiation (IR)-induced γH2AX activity, allowing for the quantification of DSB events at high spatiotemporal resolutions. Taken together, we provide a new experimental tool to evaluate the spatiotemporal dynamics of DNA double-strand breaks. Ultimately, our biosensor can be useful for elucidating the molecular mechanisms underlying DNA damage and repair processes.

**Graphical abstract:**

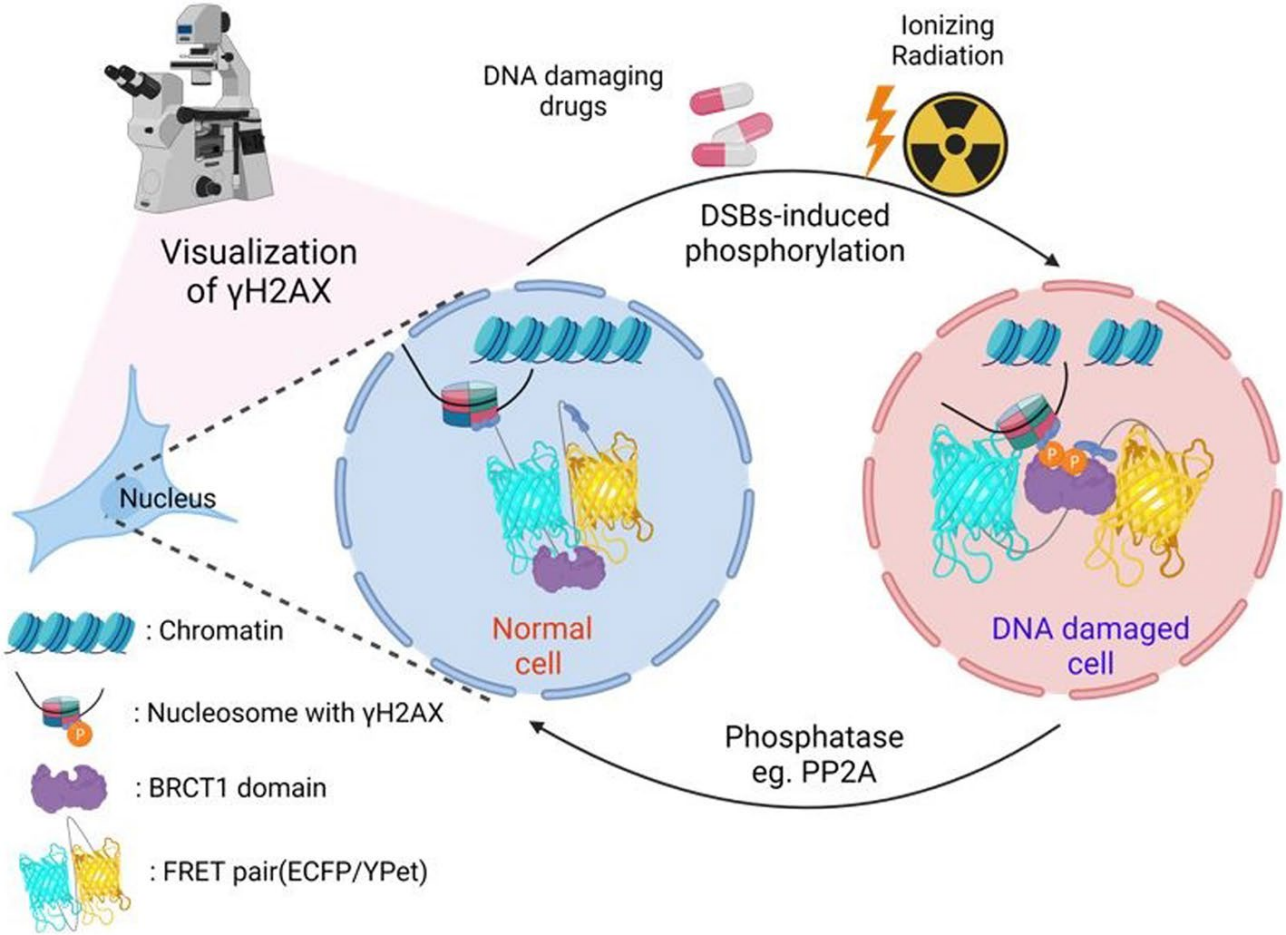

**Supplementary Information:**

The online version contains supplementary material available at 10.1186/s40824-023-00354-1.

## Introduction

DNA double-strand breaks (DSBs) are the most cytotoxic form of DNA damage within cells, leading to various detrimental consequences, including cell cycle transition, apoptosis, and mutations caused by DNA repair [1, 2]. In response to DSBs, cells trigger a complex posttranslational network known as DNA damage response (DDR). When the DDR pathway is initiated, the phosphorylation of the histone H2A variant H2AX forms at the site of DNA damage in the nucleosome [1]. The H2AX accounts for approximately 10%-15% of the H2A family [3] and the C-terminus of H2AX has a highly conserved serine residue. H2AX can be phosphorylated on Ser139 termed γH2AX in the presence of DNA damage, which has been used as a major indicator of DSB detection since it is the first step during the DNA damage and repair process. Based on previous studies, the phosphorylation of H2AX is regulated by three PI3K-related protein kinases such as ataxia telangiectasia mutated (ATM), ATM and Rad3-related (ATR), and DNA-dependent protein kinase catalytic subunit (DNA-PKcs) [4–9]. However, less is known about the molecular mechanisms involved in DNA damage and repair processes.

DDR factors contain single or multiple BRCA1 carboxy-terminal (BRCT) domains that bind to phosphorylated peptides, thereby participating in DNA repair and damage checkpoints [10, 11]. NFBD1/MDC1, a DNA damage checkpoint mediator, directly binds to γH2AX to form a DDR complex through hydrogen bonding between phosphor-Ser139 of H2AX and Arg1933 of MDC1 [6, 12]. BRCT or fork-head associated (FHA) domain-containing proteins, such as 53BP1, BRCA1, PTIP, MDC1, MRE11, RAD50, and NBS1, accumulate in foci and co-localize with γH2AX in response to DSBs [13–17]. In this way, foci are formed when DDR factors accumulate in conjunction with γH2AX, allowing for the evaluation and detection of DNA damage-induced DSBs in the nucleosome [18, 19].

Until now, antibodies against γH2AX and immunofluorescence labeling have been commonly used to detect DSBs by measuring the amount of γH2AX formed. This method has been extended to other assays, including western blot, ELISA, and 2D gel electrophoresis. In spite of the popularity of these current tools, obtaining spatiotemporal and accurate information about DSBs from living specimens is still challenging. At present, DSBs are mostly analyzed in cell lysates or fixed cells, which limits the ability to provide real-time information about the state of a specimen. Furthermore, the results from these tools can be difficult to interpret due to the complexity of the technique, and when the target protein is present at low concentrations, the signal may be weak to detect and thus may not provide the desired level of accuracy. To address this problem, we develop a genetically-encoded DSB biosensor based on fluorescence resonance energy transfer (FRET) by employing the H2AX and BRCT1 domains derived from MDC1. Ratiometric FRET biosensors are powerful tools for studying the spatiotemporal dynamics of protein–protein interactions. In this work, the biosensor developed successfully detects and visualizes DSB events, measuring γH2AX activity at high spatiotemporal resolutions in living cells. Together, our study will provide a new perspective for elucidating the molecular mechanisms involved in DNA damage and repair processes, as well as a valuable tool to develop a therapeutic approach for various pathophysiology implicated in DNA damage-induced DSBs.

## Materials and methods

### Construction of DNA plasmids

The DNA double-strand breaks biosensor (DSBS) was constructed by adding an H2AX substrate and BRCT1 domain. The BRCT1 domain from MDC1 was amplified by PCR and digested with SphI and BspEI. The H2AX substrate was the KKATQASQEY peptide (5′-TCCGGAaagaaggccacccaggcctcccaggagtacGAGCTC-3′) digested by BspEI (5′-T↓CCGGA-3′) and SacI (5′-GAGCT↓C-3′). Using two-fragment restriction cloning, the two encoding genes were fused between the N-terminal ECFP and C-terminal YPet in pRsetB (Invitrogen). The DNA insert with fluorescent proteins was digested with BamHI/EcoRI and ligated into a pcDNA3.1 vector (Invitrogen) for expression in mammalian cell lines. The wild-type DSB sensor (WT-DSBS; Fig. S1) was constructed with full-length H2AX (H2AX-FL). H2AX-FL was amplified using PCR, and digested with HindIII/BamHI. pENTR-GFP-MDC1 and pDONR223-H2AFX-WT were obtained from Addgene (plasmids #26,284 and #81,937, respectively). The H2AX substrate mutant DSB sensor (Sub-MT; Fig. S2) and H2AX-FL mutant (H2AX-MT; Fig. S3) were generated by site-directed mutagenesis, which transformed the sequence peptides into KKATQAFQEF and KKATQAAQEY of H2AX-FL, respectively. Both sites of the H2AX substrate and full-length mutant DSB sensor (Double-MT; Fig. S4) were also generated from Sub-MT by site-directed mutagenesis, which transformed the sequence peptide into KKATQAAQEY of H2AX-FL. The binding domain (BRCT1) mutant from WT-DSBS and Double-MT were generated by site-directed mutagenesis, which were called BRCT-MT (Fig. S5) and Triple-MT (Fig. S6), respectively. The amino acid of the transformed binding domain was 1933-Arg to -Gln from MDC1 [12]. pLV.ATMi was a gift from Didier Trono (plasmid #14,542; Addgene). A list of all primers used is provided in Table S1. SnapGene software was used to simulate all cloning and site-directed mutagenesis procedures.

### Cell culture, ionizing radiation, and chemicals

HEK293T cells were cultured in Dulbecco’s modified Eagle’s medium (DMEM) (CM-002; GenDepot) supplemented with 10% foetal bovine serum (FBS) (WB0015; Hyclone) and 100 U/mL penicillin and 100 µg/mL streptomycin (CA005; GenDepot). The cells were grown in a humidified incubator containing 5% CO_2_ at 37 °C in covered glass-bottom dishes (100,350; SPL). Transfection of DNA plasmids into cells was achieved using Lipofectamine 3000 (L3000015; Invitrogen) according to the manufacturer’s instructions. After transfection, the cells were exposed to X-rays using an X-ray generator (M-150WE, Softex, Tokyo, Japan) for each dose. Etoposide and fingolimod (FTY720) were purchased from Selleckchem (S1225 and S5002).

### Microscope and imaging experiment

As a means of maintaining the cells during imaging, we used a medium that was CO2-independent (18,045,088; Gibco) containing 0.5% FBS, 4 mM L-glutamine, 100 U/mL penicillin, and 100 µg/mL streptomycin. Our images were acquired with a Leica DMi8 microscope equipped with a 40X HC PL FLUOTAR L (NA 0.6 dry) and a 100X HC PL FLUOTAR (NA 1.32 oil immersion) objectives. In the ECFP, the filters consisted of 436/20 excitation filter, 455 dichroic mirror, and 480/40 emission filter, wherease in the case of FRET, it was 436/20 excitation filter, 455 dichroic mirror, and 535/30 emission filter. We used LAS X software to eliminate the background from the image and acquire ECFP, FRET, and ratio images.

### Analyzing FRET images

It was determined that the background intensity of fluorescence should be quantified by subtracting the signals from the region of interest (ROI) for FRET and ECFP channels. Based on the background-subtracted fluorescence intensity images of FRET and ECFP, the pixel-by-pixel ratio images of ECFP/FRET were calculated., as follows:

$$I\;(ECFPROI)\;-\;I\;(ECFPbg)\;/\;I\;(FRETROI)\;-\;I\;(FRETbg),$$

Where *I* represents the intensity of each channel’s region. The ratio images were displayed in intensity-modified display (IMD) mode, in which the color and brightness of the pixels were determined by the ratio of ECFP/FRET.

### Statistical analysis

All statistical analyses were performed using GraghPad7.0. Statistical data were expressed as the mean ± standard error of the mean (SEM), and statistical evaluation was performed by unpaired t-test using GraphPad 7.0. Significant differences were determined by p-values (*P < 0.05, ** P < 0.01, *** P < 0.001, and **** P < 0.0001).

## Results and discussion

### DNA double-strand breaks biosensor (DSBS) design and characterization

**Fig. 1:**
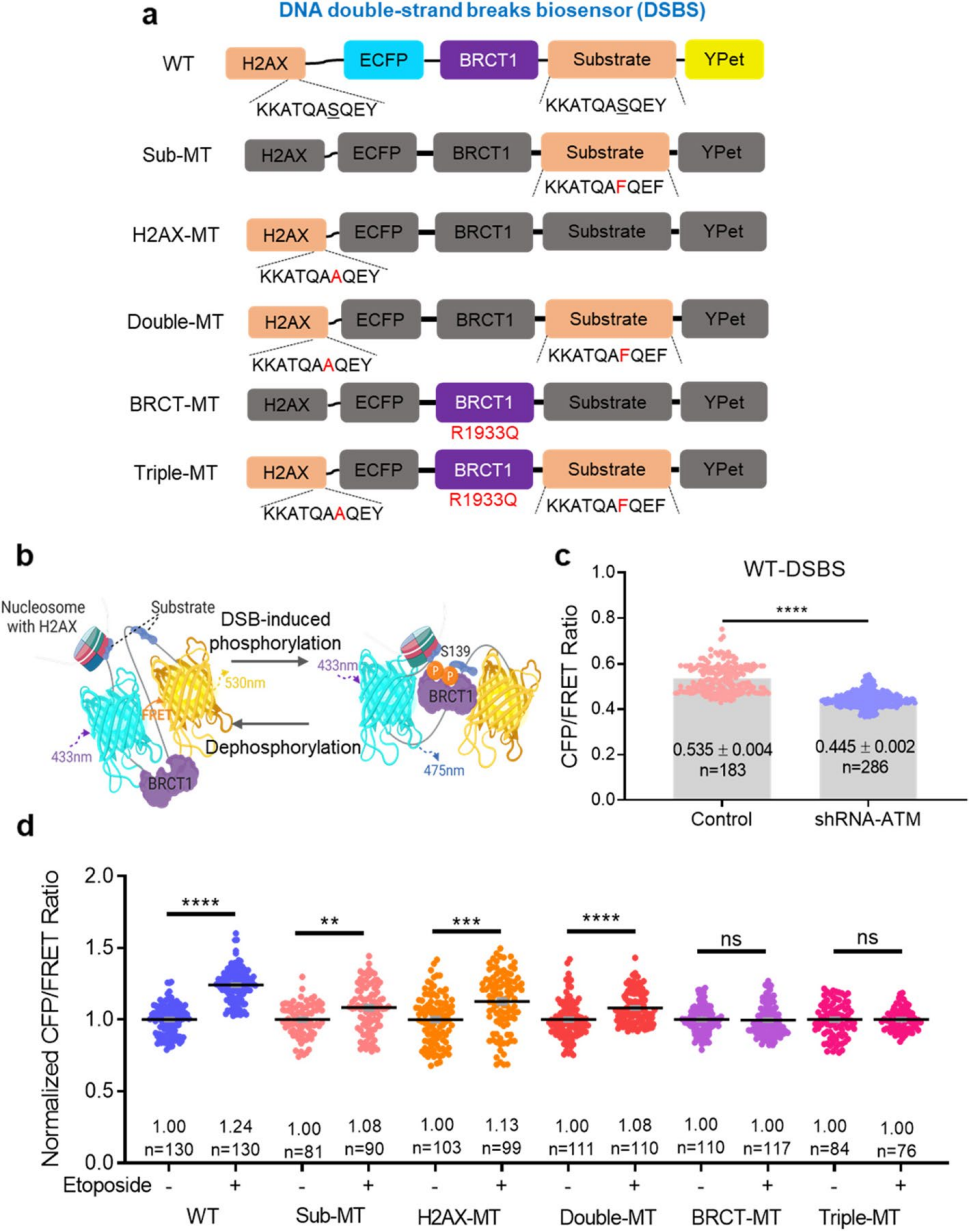
Design and characterization of the FRET-based DNA double-strand breaks biosensors (DSBS). **a** Schematic diagram of the DSBS. The DSBS is composed of full-length H2AX, an ECFP donor, BRCT1 domain derived MDC1, a substrate of partial H2AX, and an YPet acceptor. The fragments of DSBS mutants are the substrate (S139A, Sub-MT), full-length H2AX (S139F, H2AX-MT), two Ser139 phospho-peptides (Double-MT), BRCT1 (R1933Q, BRCT-MT), and the previous three residues (Triple-MT). **b** The WT-DSBS shows the conformational change by interacting between DNA double-strand breaks (DSBs)-induced phospho-Ser139 and the BRCT1 domain, leading to a decrease in the FRET. Conversely, dephosphorylation induces an increase in the FRET. This diagram was produced by BioRender (http://biorender.com/). **c** The CFP/FRET ratios of WT-DSBS in HEK293T cells transfected with control or shRNA-ATM, (5′-GATCCCCGGATTTGCGTATTACTCAGTTCAAGAGACTGAGTAATACGCAAATCCTTTTTGGAAA-3′ (sense)). **d** The normalized CFP/FRET ratios of WT-DSBS and mutants in HEK293T cells treated with 100 µM etoposide for 1 h (not significant (ns), **P < 0.01, ***P < 0.001, and ****P < 0.0001)

To assess DSB activity, we designed a FRET-based DSB biosensor (DSBS) containing full-length H2AX, ECFP, BRCT1 domain-derived MDC1, an H2AX-derived substrate (KKATQASQEY, including Ser139), and YPet (Fig. 1a). Using mutagenesis, one or all of the Ser139 peptides were substituted with Phe (F) or Ala (A), called Sub-MT, H2AX-MT, and Double-MT. A critical residue of BRCT1 from MDC1, Arg1933, elicits a strong affinity with γH2AX through a direct or indirect hydrogen bond [12]. BRCT-MT and Triple-MT were manufactured by replacing Arg1933 with Gln (Q) to prevent hydrogen bonding. Two Ser139 peptides were expected to be phosphorylated and subsequently bound to the intramolecular BRCT1 domain, causing a conformational change in the sensor (Fig. 1b). Through RNA interference, we confirmed that the CFP/FRET ratio of the wild-type DSB sensor (WT-DSBS) was significantly decreased (control: 0.535 ± 0.004, *n* = 183 vs shRNA-ATM: 0.445 ± 0.002, *n* = 286, ****p < 0.0001), indicating the inhibition of phosphorylated WT-DSBS by shRNA-ATM (Fig. 1c).

The CFP/FRET ratio of WT-DSBS treated with etoposide, a topoisomerase II inhibitor, increased by 24% compared to the group without etoposide (*n* = 130, ****P < 0.0001). On the other hand, Ser139 peptide variants (Sub-MT, H2AX-MT, and Double-MT, *n* = 81–90, *n* = 99–103, and *n* = 110–111, **P < 0.01, ***P < 0.001, and ****P < 0.0001, respectively) showed an increased rate of 8 to 13%, which is less than WT-DSBS (Fig. 1d). The reason for this increase is likely that the histone H2AX is bound to the nucleosome, causing a conformational change due to the interaction of the BRCT1 of the Sub-MT, H2AX-MT, and Double-MT with the endogenous H2AX under DNA damage stress. In addition, the heterogeneity of endogenous H2AX in the same cell line would have increased the range of ratios in Sub-MT and H2AX-MT treated with etoposide. However, neither BRCT-MT (*n* = 110–117) nor Triple-MT (*n* = 76–84) mutant sensors (BRCT1 domain mutants) showed significant changes in the CFP/FRET ratio under DNA damage stress. These results demonstrate that WT-DSBS exhibits the highest FRET sensitivity compared to mutant-DSBS and detects γH2AX increases at the single-cell level under DNA damage stress.

### Specificity of DNA double-strand breaks biosensor

Due to the lack of a tool capable of tracking real-time changes in DSBs in living cells, we applied our biosensor to this purpose. To describe the dynamic behavior of DSBs, we performed real-time FRET imaging using HEK293T cells transfected with WT-DSBS. In cells treated with etoposide, WT-DSBS increased the CFP intensity and concurrently decreased the FRET value, thus increasing the CFP/FRET ratio within the nucleus (Fig. S7). Conversely, no significant change was observed in the control group treated with DMSO (Fig. S8). As compared to the DMSO control, the ratio change increased significantly after 50 min of treatment with etoposide (Fig. S9, *n* = 4, ***P < 0.001).

**Fig. 2:**
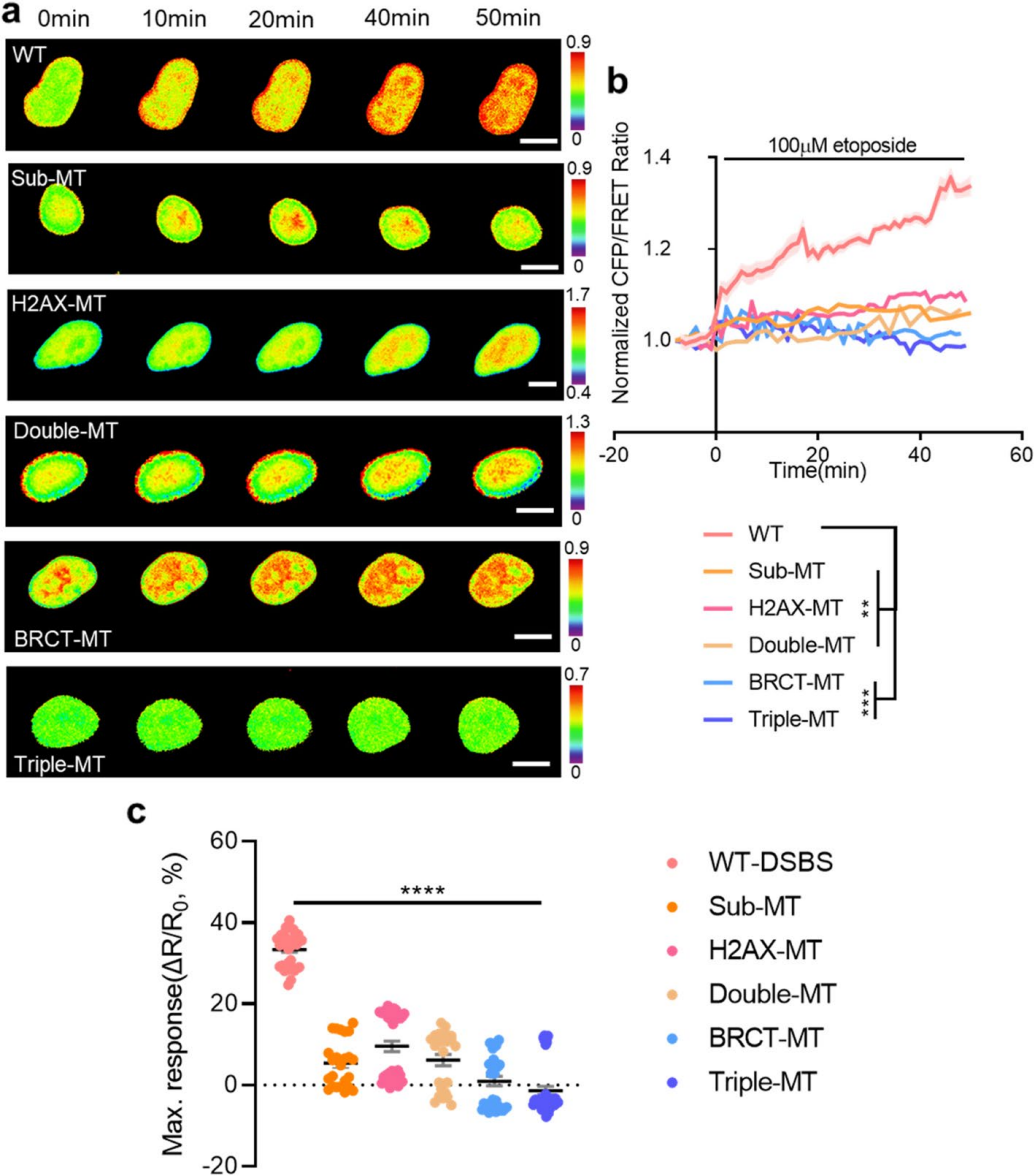
Dynamic visualization of the DSBS in response to etoposide-induced DSBs. **a** Time-lapse CFP/FRET ratio images of the WT-DSBS, Sub-MT, H2AX-MT, Double-MT, BRCT-MT, and Triple-MT in HEK293T cells treated with 100 μM etoposide. The color scale indicates high (red) and low (blue) levels of DSBs (scale bar = 10 μm). **b** Time courses of the normalized CFP/FRET ratio of the WT-DSBS (*n* = 5) and mutants (*n* = 4–5) in HEK293T cells treated with 100 μM etoposide. **c** The maximum responses of the biosensors in HEK293T cells treated with 100 µM etoposide (*n* = 24–45). The ratio changes (∆R, R_max_-R_0_) were divided by the basal ratio (R_0_), where R_max_ is the maximum ratio after etoposide treatment. All error bars represent SEM (**P < 0.01, ***P < 0.001, ****P < 0.0001)

**Fig. 3:**
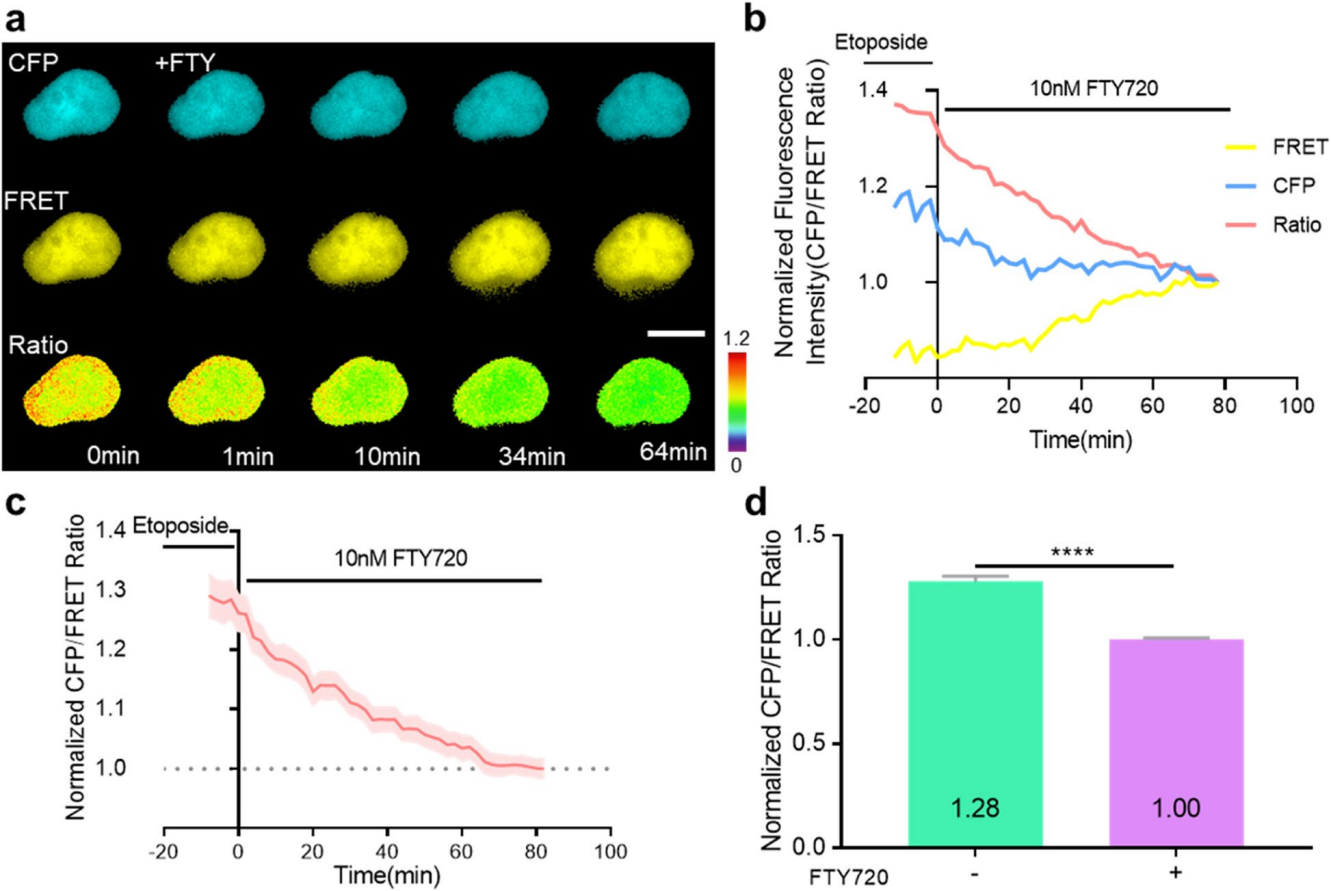
Dynamic visualization of the DSBS in response to phosphatase activation. **a** Time-lapse CFP, FRET, and CFP/FRET ratio images of the WT-DSBS in HEK293T cells pre-treated with 100 μM etoposide for 30 min and then treated with 10 nM FTY720. The color scale indicates high (red) and low (blue) levels of DSBs (scale bar = 10 μm). **b**, **c** Time courses of the normalized CFP, FRET, and CFP/FRET ratio of the WT-DSBS in HEK293T cells (d, *n* = 1; e, *n* = 4;) pre-treated with 100 μM etoposide for 30 min and then treated with 10 nM FTY720. **d** Normalized CFP/FRET ratio of WT-DSBS in HEK293T cells pre-treated with 100 µM etoposide for 30 min and then treated with 10 nM FTY720 for 1 h before (green; *n* = 15) or after (purple; *n* = 27). All error bars represent SEM (****P < 0.0001)

Next, the activity of DSB after etoposide treatment in all mutant-type biosensors manufactured in this study was also evaluated and compared. Similar to the data presented in Fig. 1d, the highest ratio change was observed in the nuclei of cells expressing WT-DSBS. A slight increase was also observed in Sub-MT, H2AX-MT, and Double-MT biosensors. In contrast, no significant change in DSB was detected in BRCT-MT and Triple-MT (Fig. 2a-b, *n* = 4–5, **P < 0.01, and ***P < 0.001). As a result of this study, WT-DSBS was found to exhibit the greatest response to γH2AX (Fig. 2c, *n* = 24–45, ****P < 0.0001). To further validate the specificity of DSBS, we used FTY720, an activator of protein phosphatase 2A (PP2A) [20, 21]. Since this drug inhibits DSB-induced phosphorylation, we expected it to decrease the CFP/FRET ratio of the WT-DSBS due to the reduction of γH2AX. Indeed, when we treated cells with FTY720 (10 nM), the CFP/FRET ratio decreased over time and returned to its basal level (Fig. 3a-d, *n* = 15–27, ****P < 0.0001). These results indicate that our WT-DSBS biosensor can effectively detect and visualize the spatiotemporal behavior of drug-induced DSB activity at the single-cell level.

### Detection of ionizing radiation (IR)-induced DSBs

**Fig. 4:**
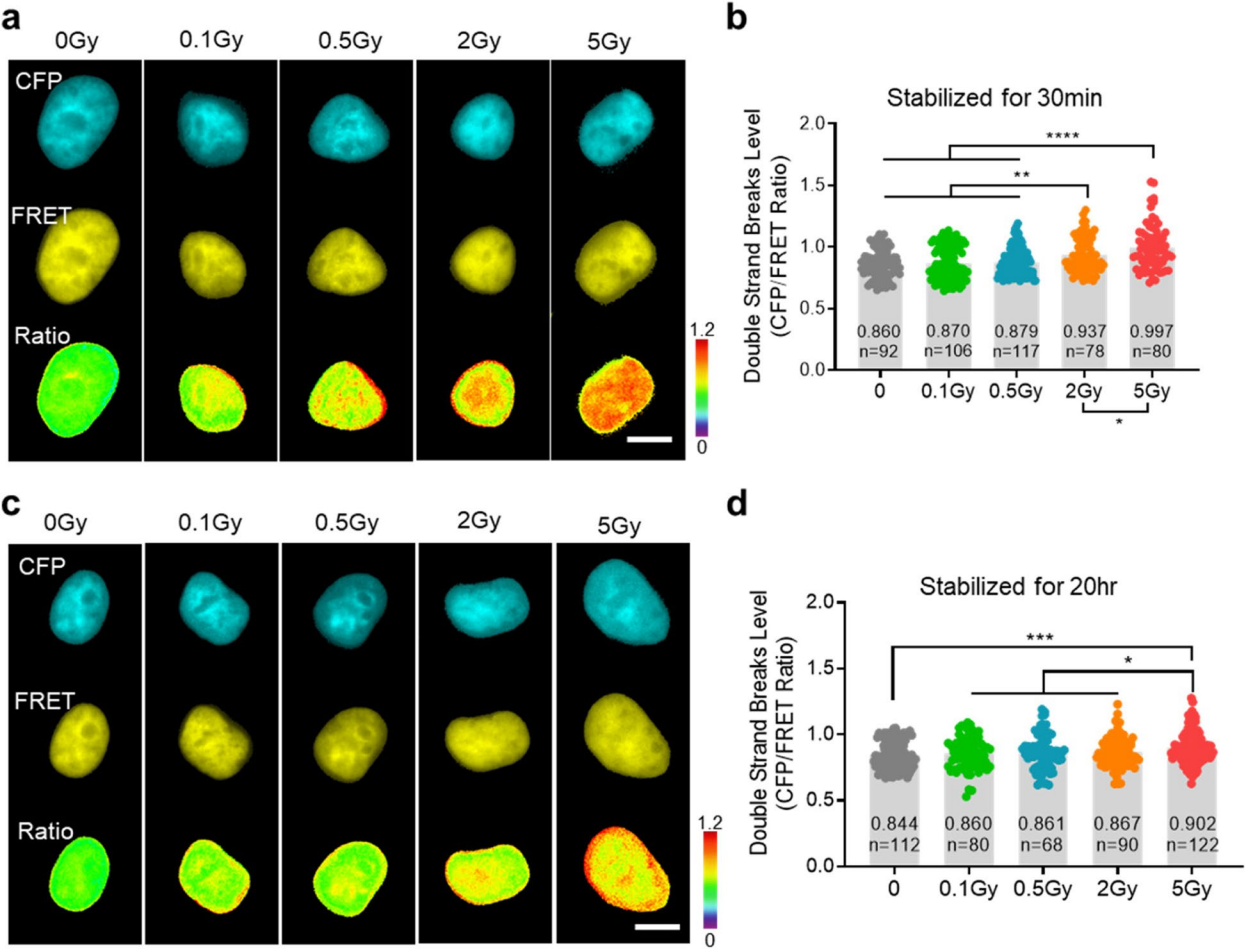
Dose-dependent detection of the WT-DSBS in response to ionizing radiation (IR). (a, c) Dose-dependent CFP, FRET, and CFP/FRET ratio images of the WT-DSBS in HEK293T cells irradiated with 0.1, 0.5, 2, or 5 Gy, and then stabilized in the incubator for 30 min (a) or 20 h (c). The color scale indicates high (red) and low (blue) levels of DSBs (scale bar = 10 μm). (b, d) Dose-dependent DSBs level using the WT-DSBS in HEK293T cells irradiated with 0.1, 0.5, 2, or 5 Gy and then stabilized in the incubator for 30 min (c) or 20 h (d). All error bars represent SEM (*P < 0.05, **P < 0.01, ***P < 0.001, ****P < 0.0001)

Radiation and chemotherapy are commonly used to treat tumors by inducing DNA damage in cancer cells [22, 23]. In general, it is well known that ionizing radiation (IR)-induced DNA damage causes DSBs. However, evidence that the extent of IR exposure causes DSBs has not been found at the single-cell level [24, 25]. In this study, the DSB activity of cells receiving different IR doses was compared and analyzed. In Fig. 4, an IR of 0.1, 0.5, 2, and 5 Gy was applied to each experimental group using an X-ray generator. After stabilization for 30 min, the DSB activity was analyzed using FRET imaging. As a result, no significant DSB-inducing effect was elicited in the 0.1 and 0.5 Gy groups (*n* = 106 and 117, respectively). In contrast, cells irradiated with 2 and 5 Gy showed a significant increase in DSB activity (Fig. 4a, b, *n* = 78, and 80, **P < 0.01, and ****P < 0.0001, respectively). In the groups treated with 0.1, 0.5, and 2 Gy of IR and stabilized for 20 h, DSB activity was restored to a level similar to that in the group not treated with IR (0 Gy). In contrast, compared with the other groups, a high level of DSBs was still detected in the 5 Gy-treated group (Fig. 4c, d, n = 122, ***P < 0.001). To our knowledge, these results are the first to highlight the change and recovery pattern of DSB activity at the single-cell level after IR administration. These findings may be helpful to determine the optimal direction of radiation therapy in various cancer cell models.

## Conclusions

Living cells are incredibly complex and dynamic systems that are constantly changing and interacting with their environment. As such, they contain a wealth of spatiotemporal information that can be used to understand the behavior of cells and their responses to external stimuli. Spatiotemporal information from living cells includes data such as the location of cellular components, the timing of cellular processes, and the movement of molecules within the cell. DNA double-strand break (DSB) is also a dynamic process that occurs when both strands of a DNA molecule are broken, and the cell must accurately rejoin the broken strands of DNA, a process that can be complex and require multiple steps. A limitation of the current tools for analyzing cell lysates and fixed cells is their inability to provide detailed information about the state of a specimen in real-time. Furthermore, these tools often produce results that are difficult to interpret due to their complexity and may not provide the desired level of accuracy when target proteins are present at low concentrations, as the signal may be weak to detect. The FRET biosensor-based imaging technique provides a powerful tool to address this challenge, as it allows for the direct visualization of DSB events in real-time. This technique is highly accurate, as it uses genetically encoded fluorescent proteins sensitive to environmental changes and provides high-resolution imaging. This tool is able to detect and measure the activity of individual molecules, allowing for a more precise understanding of the DSB processes taking place.

Additionally, this method is non-invasive and can be used to monitor the same cell over time. Furthermore, our biosensor can be used in combination with other techniques, such as microscopy, to provide even more detailed information. In practice, the DSB biosensor developed in this study was able to effectively detect and evaluate drug- and radiation-induced DSB activity. By generating and verifying the potential major types of mutant DSBS, WT-DSBS was found to exhibit the best performance. The results presented in this work provide a basis for the development of further novel quantification tools to evaluate the spatiotemporal dynamics and physiology of DNA double-strand breaks and to elucidate the molecular mechanisms of DNA damage and repair processes.

## Supplementary Information

**Additional file 1:** **Figure S1. **Plasmid map of the WT-DSBS.** Figure S2. **Plasmid map of the Sub-MT. **Figure S3. **Plasmid map of the H2AX-MT. **Figure S4. **Plasmid map of the Double-MT. **Figure S5. **Plasmid map of the BRCT-MT. **Figure S6. **Plasmid map of the Triple-MT. **Figure S7. **Time-lapse images and time courses of CFP, FRET, and the CFP/FRET ratio of the WT-DSBS in HEK293T treated with 100 μM etoposide. The colour scale indicates high (red) and low (blue) DSB levels (scale bar = 10 μm). **Figure S8. **Time-lapse images and time courses of CFP, FRET, and the CFP/FRET ratio of the WT-DSBS in HEK293T cells treated with DMSO (control). The colour scale indicates high (red) and low (blue) DSB levels (scale bar = 10 μm). **Figure S9. **Time course of the normalized CFP/FRET ratio of the WT-DSBS in HEK293T cells treated with DMSO (control, *n* = 4) or 100 μM etoposide (*n* = 4). All error bars represent the SEM (***P < 0.001).** Table S1. **Primers for cloning and site-directed mutagenesis used in this study.

## Data Availability

Data available on request from the authors.

## Abbreviations

ATM: Ataxia telangiectasia mutated
ATR: ATM and Rad3-related
bg: Background
BRCT: BRCA1 carboxy-terminal
DDR: DNA damage response
DNA-PKcs: DNA-dependent protein kinase catalytic subunit
DSBs: DNA double strand breaks
DSBS: DNA double strand breaks biosensor
FHA: Fork-head associated
FRET: Fluorescence resonance energy transfer
γH2AX: Phosphorylated Ser139 H2AX
IMD: Intensity-modified display
IR: Ionizing radiation
NA: Numerical aperture
PP2A: Protein phosphatase 2A
ROI: Region of interest
SEM: Standard error of the mean
