# Supplementary material for: A novel DNA double-strand breaks biosensor based on fluorescence resonance energy transfer
Source: Biomater Res. 2023 Feb 17;27:15. doi: 10.1186/s40824-023-00354-1 (PMC9936723; doi:10.1186/s40824-023-00354-1)
Supplement: Supplementary file 1 — Additional file 1: Figure S1. Plasmid map of the WT-DSBS. Figure S2. Plasmid map of the Sub-MT. Figure S3. Plasmid map of the H2AX-MT. Figure S4. Plasmid map of the Double-MT. Figure S5. Plasmid map of the BRCT-MT. Figure S6. Plasmid map of the Triple-MT. Figure S7. Time-lapse images and time courses of CFP, FRET, and the CFP/FRET ratio of the WT-DSBS in HEK293T treated with 100 μM etoposide. The colour scale indicates high (red) and low (blue) DSB levels (scale bar = 10 μm). Figure S8. Time-lapse images and time courses of CFP, FRET, and the CFP/FRET ratio of the WT-DSBS in HEK293T cells treated with DMSO (control). The colour scale indicates high (red) and low (blue) DSB levels (scale bar = 10 μm). Figure S9. Time course of the normalized CFP/FRET ratio of the WT-DSBS in HEK293T cells treated with DMSO (control, n = 4) or 100 μM etoposide (n = 4). All error bars represent the SEM (***P < 0.001). Table S1. Primers for cloning and site-directed mutagenesis used in this study. [file 40824_2023_354_MOESM1_ESM.docx]

**Supplementary Material**

**A novel DNA double-strand breaks biosensor based on fluorescence resonance energy transfer**

Jung-Soo Suh ^a^ and Tae-Jin Kim ^a,b,c^*

^a^Department of Integrated Biological Science, Pusan National University, Pusan 46241, Republic of Korea; ^b^Department of Biological Sciences, Pusan National University, Pusan 46241, Republic of Korea; ^c^Institute of Systems Biology, Pusan National University, Pusan 46241, Republic of Korea

***Corresponding Author:**

Tae-Jin Kim, Ph.D.

Department of Biological Sciences, Pusan National University, Pusan 46241, Republic of Korea; Tel: 82-51-510-2261; Fax: 82-51-581-2962; E-mail: [tjkim77@pusan.ac.kr](mailto:tjkim77@pusan.ac.kr)

**Figure S1-S9**

**Table S1**


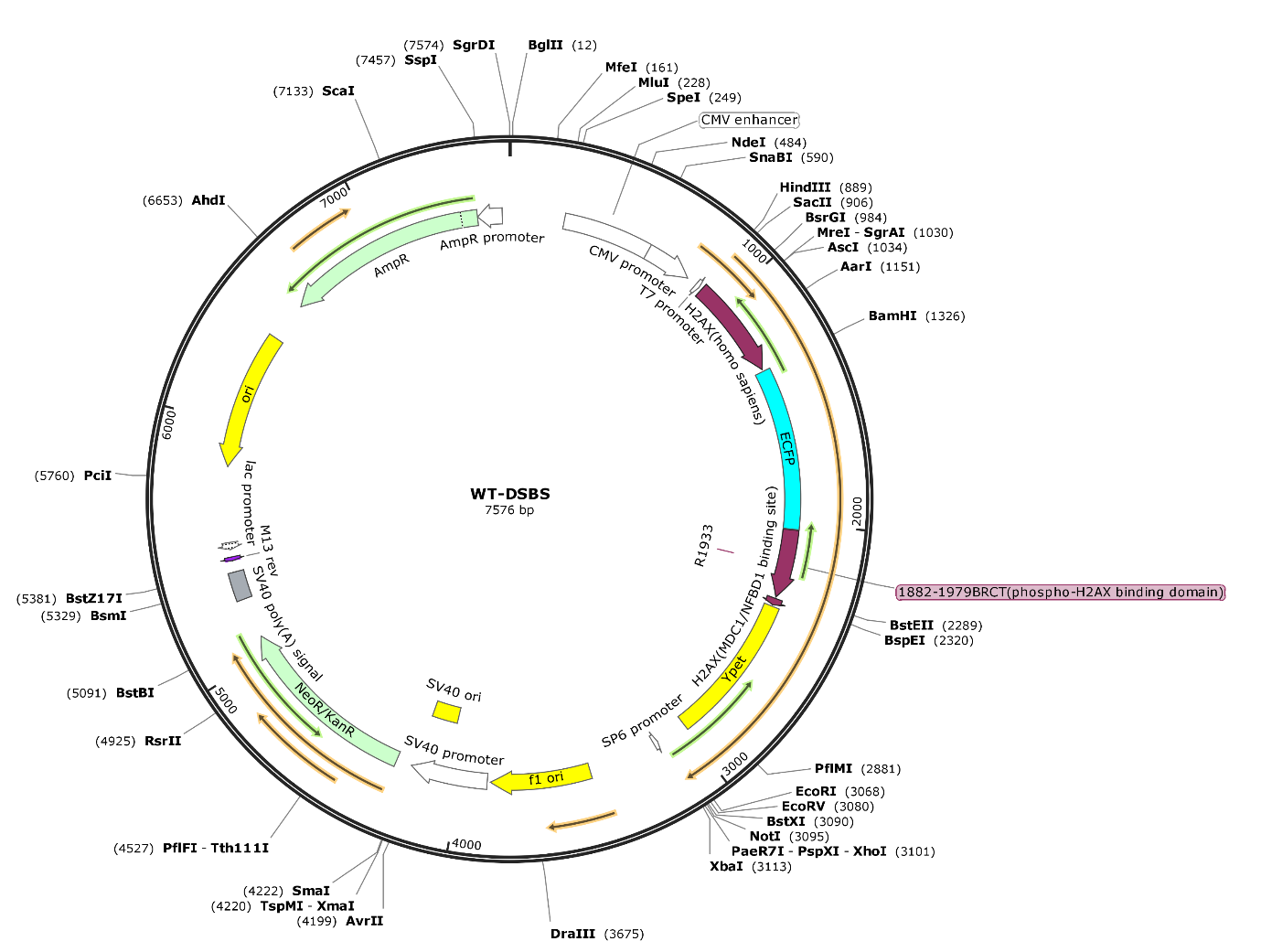


**Figure S1.** Plasmid map of the WT-DSBS.

**Figure S2.** Plasmid map of the Sub-MT.


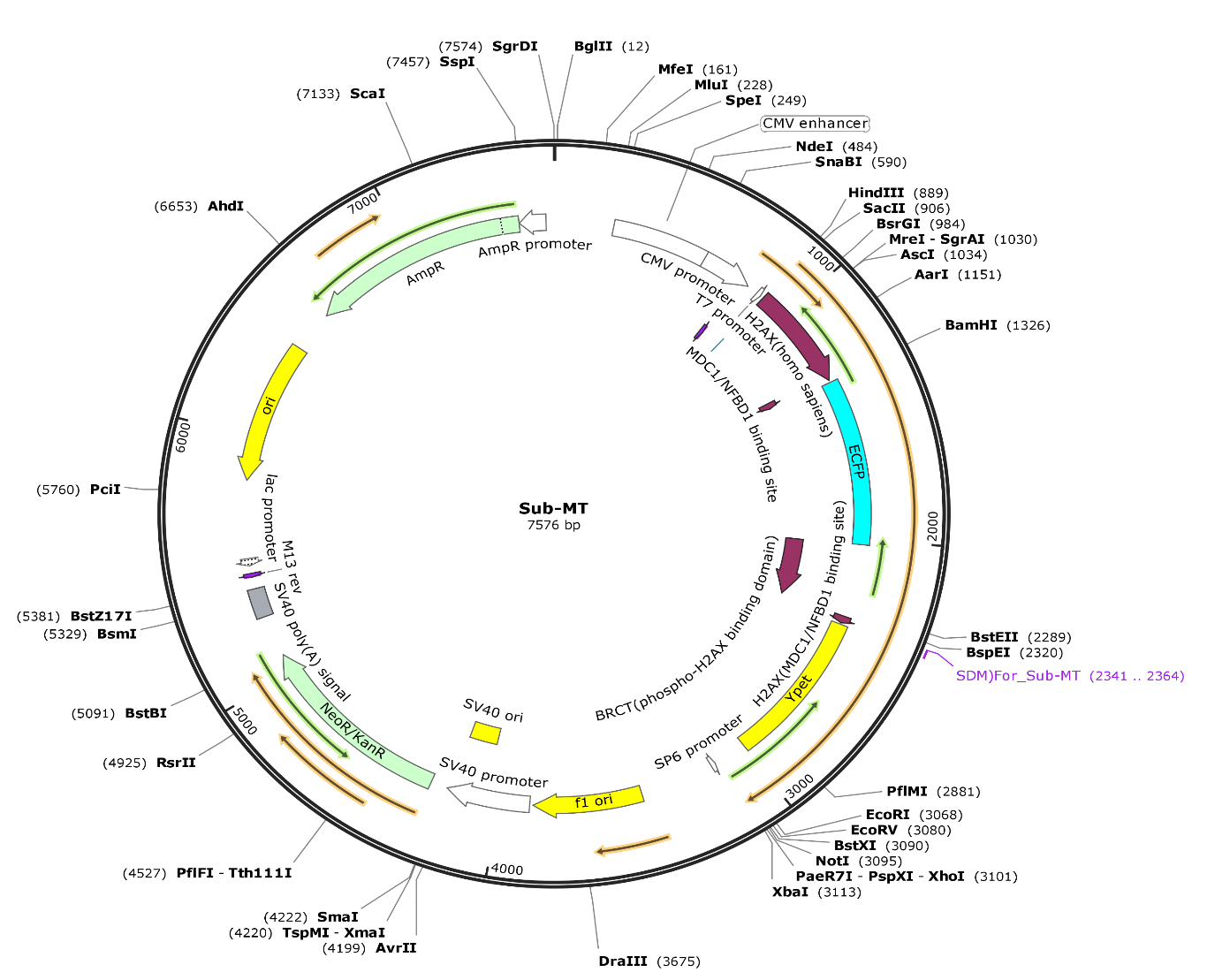

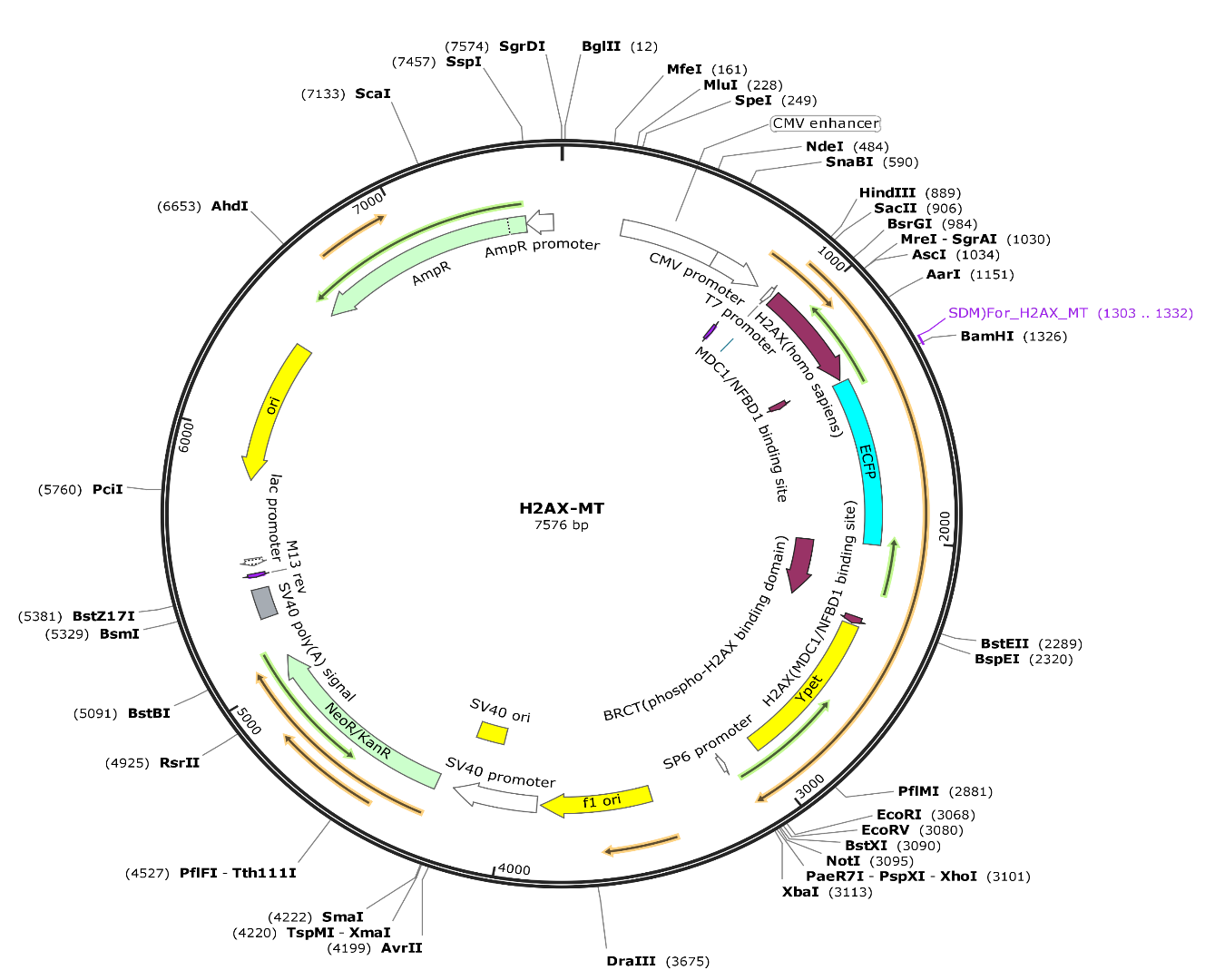


**Figure S3.** Plasmid map of the H2AX-MT.


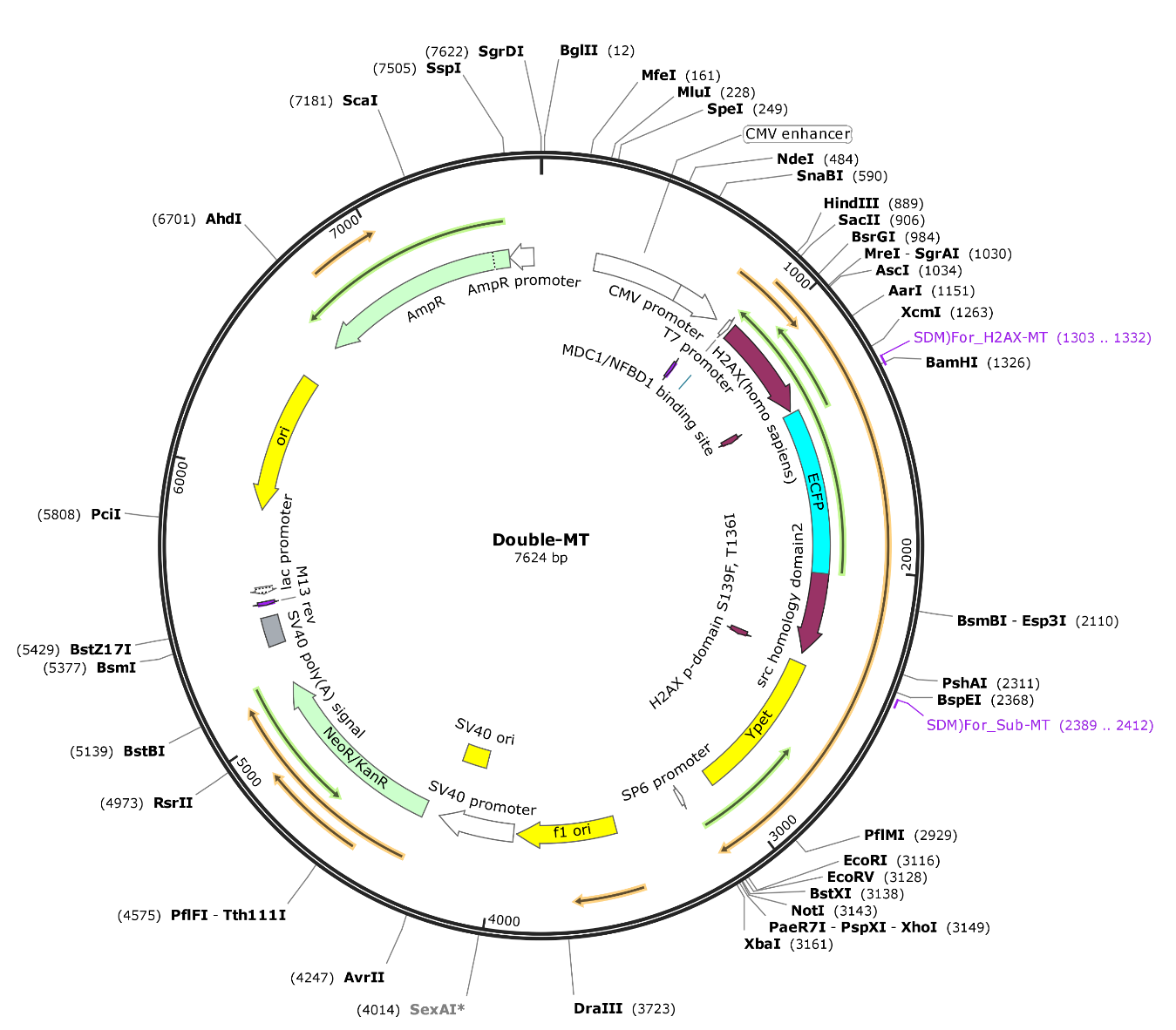


**Figure S4.** Plasmid map of the Double-MT.


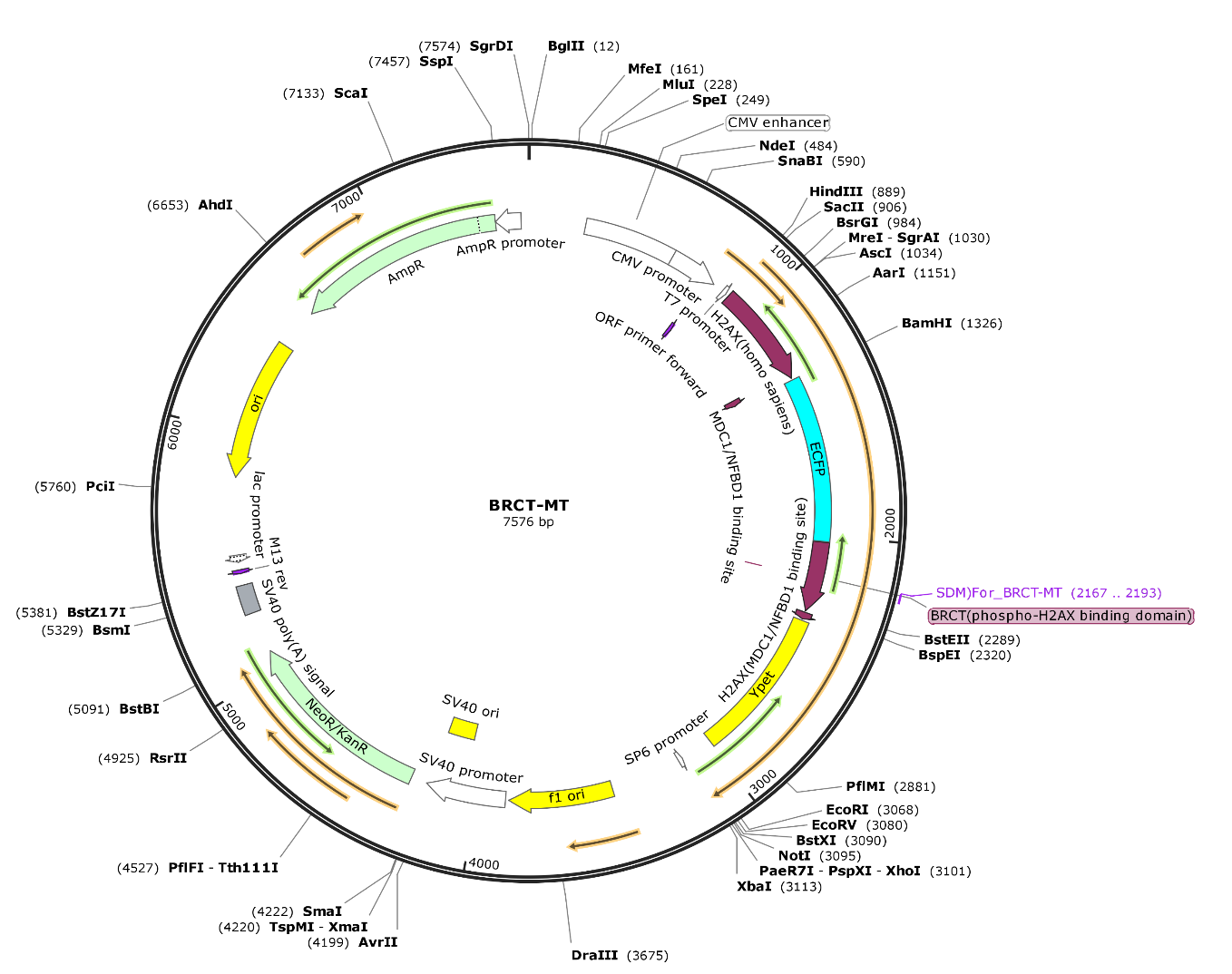


**Figure S5.** Plasmid map of the BRCT-MT.


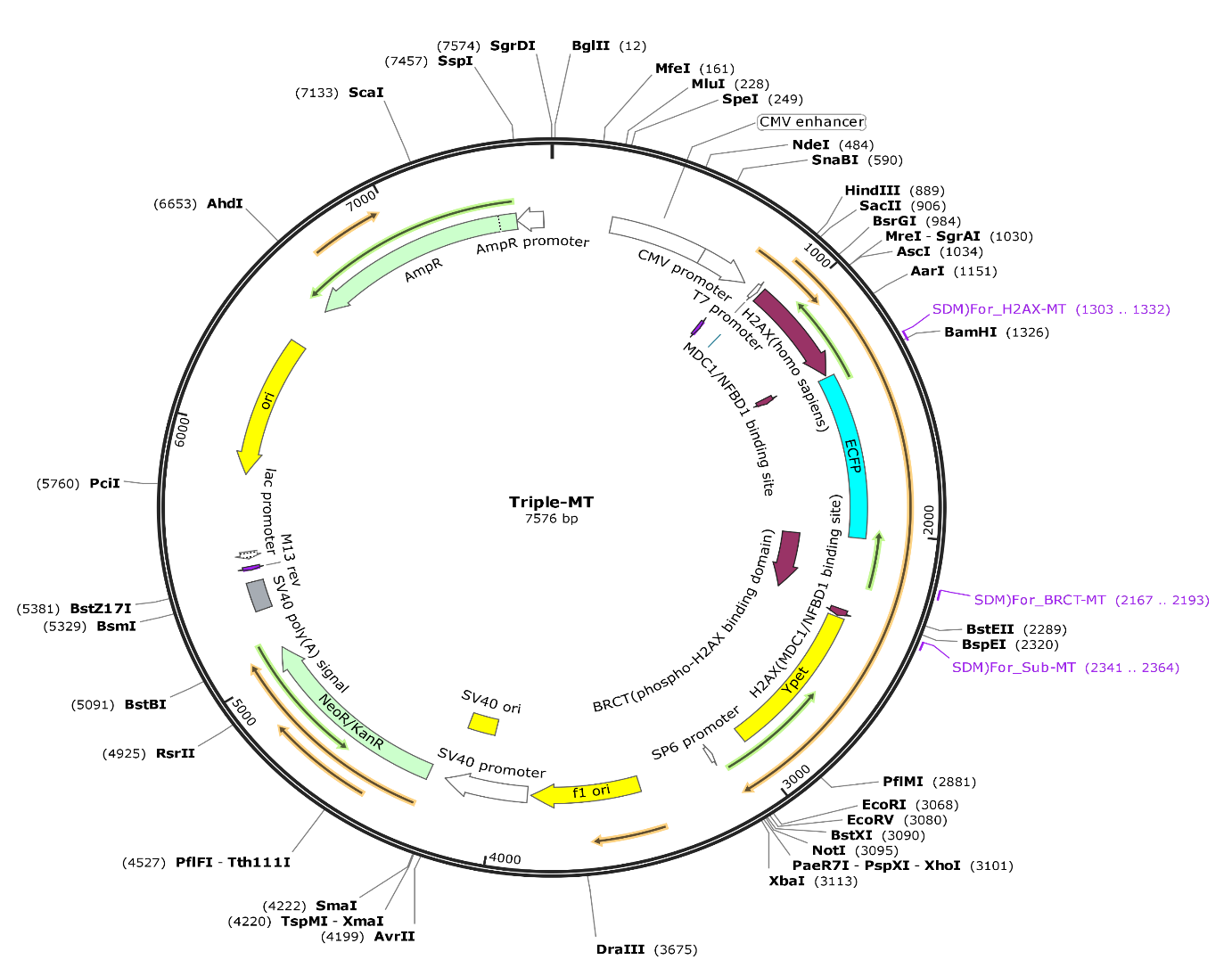


**Figure S6.** Plasmid map of the Triple-MT.


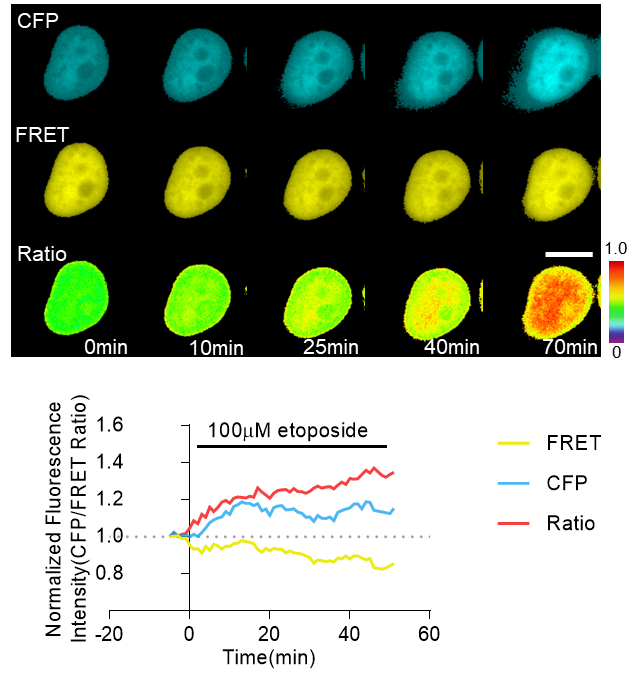


**Figure S7.** Time-lapse images and time courses of CFP, FRET, and the CFP/FRET ratio of the WT-DSBS in HEK293T treated with 100 μM etoposide. The colour scale indicates high (red) and low (blue) DSB levels (scale bar = 10 μm).


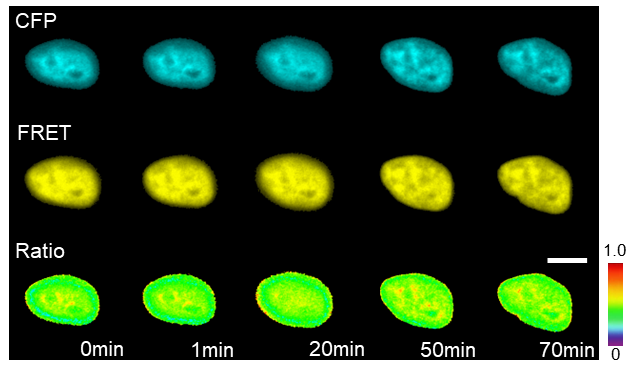

**Figure S8.** Time-lapse images and time courses of CFP, FRET, and the CFP/FRET ratio of the WT-DSBS in HEK293T cells treated with DMSO (control). The colour scale indicates high (red) and low (blue) DSB levels (scale bar = 10 μm).

**Figure S9.** Time course of the normalized CFP/FRET ratio of the WT-DSBS in HEK293T cells treated with DMSO (control, n = 4) or 100 μM etoposide (n = 4). All error bars represent the SEM (****P* < 0.001).

**Table S1.** Primers for cloning and site-directed mutagenesis used in this study

| Primers | Sequence (5'-3') |
| --- | --- |
| BRCT_Forward | CAATGCATGCAACGACGGACCAAACTTAA |
| BRCT_Reverse | GCATTCCGGAAAAGTTCTTCTCTTGCTC |
| H2AX_Forward | ACCCAGGCCTTCCAGGAGTTCATGGATCCC |
| H2AX_Reverse | GGGATCCATGAACTCCTGGAAGGCCTGGGT |
| Sub-MT_Forward | GCCTTCCAGGAGTTCGAGCTCATG |
| Sub-MT_Reverse | CATGAGCTCGAACTCCTGGAAGGC |
| H2AX-MT_Forward | CAGGCCGCCCAGGAGTACATGGATCCC |
| H2AX-MT_Reverse | GGGATCCATGTACTCCTGGGCGGCCTG |
| BRCT-MT_Forward | GATCGCATCCGCCAGACAGTCAAGTTC |
| BRCT-MT_Reverse | GAACTTGACTGTCTGGCGGATGCGATC |
